# S01-2 Providing policymakers with the evidence of the true value of physical activity to individuals and communities

**DOI:** 10.1093/eurpub/ckac093.003

**Published:** 2022-08-29

**Authors:** Chris Scott

**Affiliations:** Communications, London Sport, London, United Kingdom

**Keywords:** Social value, policymakers, data and insight, collaboration

## Abstract

**Background:**

With many countries and communities suffering from budget cuts, policymakers are coming under increasing pressure to provide the true value of public health interventions. Owing to the scale of conclusive findings over the past decade, we now have a robust evidence base regarding the impact of physical activity on individuals and communities. However, more work needs to be done to bridge the gap between policymakers and academics.

**Methods:**

We conducted both a review of the leading academic research as well as primary analysis of existing open-access datasets to determine the impact of physical activity on the risk of health and social outcomes across the life course. This evidence base was then modelled with the latest figures on disease prevalence and its economic impact to quantify the social and economic value of physical activity.

**Results:**

This methodology has currently been applied to a city population, as well as a national scale evaluation of the impact of individual sports on children alone. Results have shown that the average value of physical activity, across four cities around the world including London, Stockholm, Singapore and Auckland, is US$1900 per person in economic, health and social outcomes. In addition to this when looking specifically at the 4 million children who participate in team sports across England, preliminary findings estimate the total value to be £6.5Bn annually.

**Conclusions:**

These initial findings are already being used by policymakers to make the case for additional investment and change the way they evaluate the outcome of their policies and programmes. For example, Aktive Auckland has secured an additional NZ$120M for sport and recreation in the 10-year annual national budget after they used results which showed the annual contribution of physical activity was $1.9Bn to the local economy. Sports federations in the UK are also reshaping their strategies towards childhood sport and activity based on the emerging findings of the report.

